# Semaphorin 3F induces colorectal cancer cell chemosensitivity by promoting P27 nuclear export

**DOI:** 10.3389/fonc.2022.899927

**Published:** 2022-09-02

**Authors:** Miaomiao Tao, Hongbo Ma, Xiaoyuan Fu, Cancan Wang, Yanyan Li, Xiaoqiao Hu, Renming Lv, Gendou Zhou, Jun Wang, Ruyan Liu, Meiyu Zhou, Guofa Xu, Zexin Wang, Xiurong Qin, Yi Long, Qunzhen Huang, Min Chen, Qi Zhou

**Affiliations:** Department of Oncology, Fuling Central Hospital of Chongqing City, Chongqing, China

**Keywords:** Semaphorin 3F, colorectal cancer, chemosensitivity, p27, degradation

## Abstract

Colorectal adenocarcinoma (CRC) is the third most common malignancy worldwide. Metastatic CRC has a poor prognosis because of chemotherapy resistance. Our previous study demonstrated that semaphorin 3F (SEMA3F) signaling may contribute to reversing chemotherapy resistance in CRC cells by reducing E-cadherin and integrin αvβ3 expression levels. Another study showed that upregulation of p27 significantly increase the expression of E-cadherin and integrin. This study aimed to evaluate the effect of SEMA3F on P27 and whether it can reverse resistance in CRC cells. We compared the chemosensitivity of human colorectal cancer cell lines with different SEMA3F expression levels to 5-Fu through cell experiment and animal experiment. Then the interaction between SEMA3F and p27 and its possible mechanism were explored by Western Blot, immunofluorescence and immunocoprecipitation. We also compared the disease-free survival of 118 CRC patients with high or low expression of SEMA3F.The results showed that overexpresstion of SEMA3F enhanced the chemotherapy sensitivity and apoptosis of CRC cells *in vitro* and *in vivo*. Among 118 postoperative CRC specimens, the disease-free survival of patients with positive SEMA3F expression was significantly longer than that with negative SEMA3F expression after adjuvant treatment. Upregulation of SEMA3F in multicellular spheroid culture (MSC) could increase p27 phosphorylation at serine 10 (Ser10), subsequently promote the cytosolic translocation of P27. Overall, our results reveal a novel molecular mechanism: SEMA3F mediates the degradation of p27 and regulates its subcellular localization to enhance chemosensitivity to 5-Fu in CRC cells, rather than inhibits p27 expression.

## Introduction

Colorectal adenocarcinoma (CRC) is the third most common malignancy worldwide (1, 2), and the prognosis of metastatic CRC (mCRC) remains poor due to chemotherapy resistance. *In vivo*, solid tumors are three-dimensional (3D) spherical blocks,which depend on cell-cell adhesion and cell-matrix interactions (3, 4). In contrast to resistance in two-dimensional (2D) cultures of tumor cells *in vitro*, multicellular resistance (MCR) *in vivo* is caused by cell adhesion within tumors to avoid anoikis (5). Simultaneously, cell-cell contact and extracellular matrix (ECM) attachments prompt tumor cells to enter quiescence or predominantly the G1 phase of the cell cycle to induce chemotherapy resistance (6–8). E-cadherin, a member of the classical cadherin family, is widely expressed in epithelial cells, including the cells that make up various types of epithelial cancers (9). It plays a key role in regulating adhesion in multicellular spheroid cultures (MSCs) of tumor cells (10–12). p27, a cyclin-dependent kinase inhibitor, is upregulated in multicellular spheroid tumor cells. Previous studies have demonstrated that increased cell-cell adhesion in MSCs affects cell cycle regulation by increasing p27 levels (13, 14). Therefore, p27 is a key regulator of contact-dependent growth inhibition that induces cytotoxic drug resistance in multicellular spheroids by increasing E-cadherin and integrin expression.

Previous studies have shown that semaphorin 3F (SEMA3F), a class 3 semaphorin protein, is a suppressor gene located on chromosome 3p21.3 (15). Many studies have also suggested that SEMA3F, the inhibitory ligand of NRP2, has a repressive effect on growth, angiogenesis, lymphangiogenesis and invasion in several cancers (16–19). We previously reported that the loss of SEMA3F contributed to CRC cell metastasis (20). Furthermore, our previous study demonstrated that SEMA3F expression significantly reduced αvβ3 integrin expression in CRC cells to reverse chemotherapy resistance (21). However, p21 and p27 inhibited anchorage-dependent growth to induce chemotherapy resistance *via* the upregulation of adhesion molecule expression (7, 10, 22–24). VEGF-induced cell cycle progression, including the upregulation of cyclin D1, was reduced by SEMA3A (25), suggesting that SEMA3F may be implicated in cell cycle progression. We thus hypothesize that SEMA3F signaling potently inhibits p27 to decrease the effects of E-cadherin and integrin and contribute to reversing CRC MCR.

MSC can mimic the anchorage-dependent growth that tumor cells use to survive, display strong adhesion of multicellular spheroids, and can be used to generate powerful *in vitro* models to perform preclinical chemosensitivity assays (26). To explore the hypothesis that SEMA3F inhibits p27 to reverse MCR in CRC cells, we analyzed whether SEMA3F plays an underlying role in enhancing multicellular chemosensitivity to 5 fluorouraci (5-Fu) in CRC cells. Here, we report that CRC patients with SEMA3F expression exhibited an improved objective clinical response to 5-Fu. Overexpression of SEMA3F induced the apoptosis of CRC cells in MSCs exposed to 5-Fu and chemosensitivity to 5-Fu in transplanted tumors. SEMA3F inhibited tumor cell proliferation and promoted apoptosis by inducing p27 phosphorylation at Ser10 to promote p27 nuclear export. Our findings demonstrate for the first time that SEMA3F plays a novel role and mediates the mechanism of chemosensitivity to 5-Fu in CRC.

## Materials and methods

### Tissue samples

118 CRC postoperative tissue specimens were collected at Fuling Central Hospital of Chongqing City. All cases were diagnosed by histopathologists according to the Union for International Cancer Control (UICC) classification system. According to the TNM staging criteria for colorectal cancer in the seventh edition of the American Joint Committee (AJCC), all operative patients were defined as stage II to III and had received 5-Fu-based adjuvant chemotherapy for 3-6 months, with an average treatment time of 4.5 ± 1.5 months. All CRC patients had received 4 to 6 cycles of 5-Fu combination chemotherapy. The tissues were fixed in 10% formaldehyde and embedded in paraffin for histological sectioning. All human experiments were carried out in accordance with the Declaration of Helsinki (2008) of the World Medical Association and were approved by the Ethics Committee of Fuling Central Hospital of Chongqing City.

### Cell culture

The human colon cancer cell lines HCT-116, LS174T, HT-29, SW480, SW620, LoVo and RKO were obtained from the American Type Culture Collection (ATCC, Manassas, VA) and maintained in L-15 (Invitrogen Corp.) supplemented with 10% fetal bovine serum at 37°C under 5% CO_2_. CRC cell spheroids were formed using a 3D culture technique (21). Briefly, the cells were seeded in 24-well plates coated with 2% SeaPlaque agarose (BioWhittaker Molecular Applications, Rockland, Maine, USA) at 5×10^4^ cells/well in 500 μl of DMEM.

### Antibodies and reagents

Monoclonal anti-B-cell lymphoma-2 (Bcl-2, #sc-7382), anti-phospho-Bcl-2-Ser70 (# sc-293128), anti-BCL-XL (#sc8392), anti-p27 (#sc-393380), anti-MRP (#sc-365635), and anti-MCL-1 (#sc-69840) antibodies were obtained from Santa Cruz Biotechnology (Santa Cruz, CA, USA). Anti-actin antibody (#sc-8432), used as a control for western blotting (WB), was purchased from Santa Cruz Biotechnology (Heidelberg, Germany). Anti-phospho-Bcl-2-Thr56 (#2875) was purchased from Cell Signaling Technology (Denver, MA, USA). Anti-phospho-p27-Ser10 antibody (#346300) was purchased from Invitrogen. Rabbit polyclonal anti-SEMA3F (#AB5471P) was obtained from Chemicon International (Temecula, USA), and rabbit anti-cleaved Caspase3 (#9664) and anti-Caspase3 (#9662) were purchased from Cell Signaling Technology (Boston, MA, USA; 1:100). Anti-kinase-interacting stathmin (KIS) antibody (#AJ1743c) was obtained from Abgent (San Diego, CA, USA). HRP-conjugated secondary antibodies were purchased from Cell Signaling Technology (Beverly, MA, USA).

### Immunohistochemical and immunofluorescence microscopy

All tissue sections were routinely dewaxed, rehydrated, and prepared for immunohistochemistry. The slides were incubated in 0.3% H_2_O_2_ in methanol for 30 min to block endogenous peroxidase. Antigens were retrieved with 10 mmol/L sodium citrate (pH=6) for 15 min in a microwave oven. The slides were then incubated with the selected antibody at 37°C for 1 h and at 4°C overnight. Slides without primary antibody treatment served as negative controls. The slides were developed with the EnVisionTM method (Dako, Capinteria, CA), visualized using diaminobenzidine solution (Dako, Capinteria, CA) and then lightly counterstained with hematoxylin. Immunohistochemical staining was scored from 0 to 4 as follows: no staining = 0, weak staining = 1, strong staining of 25% or moderate staining of <80% of the tumor cells = 2, strong staining of 25-50% or moderate staining of >80% of the tumor cells = 3, and strong staining of >50% of the tumor cells = 4. Moreover, samples with a score from 0-1 were defined as negative (recorded as −), and those with a score from 2-4 were defined as positive (2 recorded as +, 3 as ++, 4 as +++). Ten representative areas were assessed from high-power fields for each tissue section. The slides were examined and scored independently by 3 pathologists blinded to other patient information.

Samples for immunofluorescence staining were fixed in ice-acetone for 20 min, washed with PBS 3 times for 5 min each, and incubated for 30 min at room temperature in a protein-blocking solution. The sections were incubated with the primary antibodies for 1 h at 37°C and then at 4°C overnight. After washing, the sections were incubated at 37°C for 1 h with the appropriate secondary antibodies, including FITC-conjugated goat anti-rabbit IgG (1:50, Santa Cruz), FITC-conjugated goat anti-mouse IgG (1:50, Santa Cruz), and TRITC-conjugated goat anti-mouse IgG (1:50, Beyotime, China). The sections were counterstained with Hoechst 33258 to reveal the cell nuclei.

### Transfection plasmids and establishment of stable cell lines

The SEMA3F expression vector pSectag-SEMA3F was kindly provided by Dr. David Ginty and used as described previously (16). SEMA3F RNAi expression vectors and control plasmids were obtained and used as previously described (20). Small interfering RNAs (siRNAs) against human p27 (#sc-270646, p27 siRNA: 5′-AAGCACUGCCGAGAUAUGGAAUU-3′) and a scrambled siRNA (ctrl-siRNA, 5′-AAGACCGAGCCAUUGAGGUAAUU-3′) were obtained from Santa Cruz Biotechnology in their deprotected and desalted forms. KIS siRNA and control plasmids were purchased from Sigma-Proligo, and siRNAs against human KIS were obtained from Ambion (Austin, TX) in their deprotected and desalted forms. The chemically synthesized, double-stranded siRNA contained 21-nucleotide duplex RNA sequences targeting human KIS mRNA (sense, 5′-AAGCAGUUCUUGCCGCCAGGA-3′), and vectors containing these sequences were constructed as described previously (24). All resultant constructs were verified by DNA sequencing and then transfected into target cells with Lipofectamine™ 2000 transfection reagent (Invitrogen, Carlsbad, CA, USA). Transfected cells were enriched by 1 week of antibiotic selection.

### Protein extraction and WB

Cell lysates were prepared with M-PER™ Mammalian Protein Extraction Reagent (Pierce, PA, USA). Nuclear lysates were prepared with NE-PER™ Mammalian Protein Extraction Reagent (Pierce, PA, USA). Lysates containing a total of 30 μg of protein were separated by SDS-PAGE after heat denaturation, transferred onto PVDF membranes, and incubated with 5% nonfat milk dissolved in PBS-Tween 20 for 1 h, followed by incubation with a primary antibody overnight at 4°C. After washing, the membranes were incubated with the appropriate HRP-conjugated secondary antibody and then developed with enhanced chemiluminescence (ECL) detection reagents (Amersham Pharmacia Biosciences).

### Coimmunoprecipitation assay

Total protein lysates (500 μg) from each sample were immunoprecipitated in 400 μL of lysate buffer containing 2 μL of anti-SEMA3F antibody and inhibitors of various proteases, phosphatases and kinases at 4°C for 4 h with rotation. Protein A-conjugated agarose beads (25 μL) were then added to the immunoprecipitation reaction and incubated for an additional 5 h at 4°C with rotation. The antigen–antibody complexes were precipitated by quick centrifugation, followed by 4 washes with cold PBS. For controls, the immunoprecipitation reaction included an aliquot of rabbit serum instead of the anti-SEMA3F antibody. The pellets were suspended in 20 μL of 2× SDS reducing WB loading buffer and boiled for 10 min, followed by SDS-PAGE analysis. The SEMA3F immunoprecipitates were subjected to WB assays to detect SEMA3F and P27 in the immunoprecipitates.

### Chemotherapeutics and chemosensitivity assays

Cells were plated in monolayer cell culture (MC) and MSC for 48 h in complete media as described above.Then chemotherapeutic agent (5-Fu) was added to the desired final concentration (10,50,100,200 and 400umol/L) in the medium for 48 h. The cells were then harvested, rinsed in PBS to remove residual drug, placed in 0.1% trypsin-EDTA (Life Technologies, Inc./Invitrogen) in PBS for 10-15 min at 37°C (to disaggregate intact spheroids) and then replated as follows. 10,000 cells/well (5 wells/group) were performed in 100 μL of complete medium in 96-well plates. 20 μL of 3-(4,5-dimethylthiazol-2-yl)-2,5-diphenyltetrazolium bromide (MTT) were added to each well at a final concentration of 5mg/mL. After 3 h of reaction, the reagent was removed, and 150 μL DMSO was added to dissolve the product. The plates were read using a enzyme-linked immunometric meter at a wavelength of 490 nm. The half-maximal inhibitory concentration (IC50) was defined as the concentration of 5-Fu that inhibited cell viability by 50%.

### Apoptosis assay

Immediately following 5-Fu treatment (100 μmol/L) in 3D cultures as described above, the cells were harvested (and then kept on ice), rinsed in PBS, incubated in 0.1% trypsin-EDTA in PBS for 10-15 min at 37°C, resuspended in complete medium to inactivate trypsin, rinsed twice in PBS, and fixed in cold buffer for 15 min. The fixed cells were then rinsed twice in buffer and stained with 50 µg/ml propidium iodide (Sigma Chemical Co., Mississauga, ON, Canada) in PBS for 15 min while protected from light and then analyzed using a FACSCalibur analyzer (BD Biosciences, San Jose, CA). The proportion of apoptotic cells was detected by Annexin V/7AAD staining using an Annexin V-PE Apoptosis Detection Kit (BD Biosciences). HT-29 cells in 2D culture were used as the control group. HT-29 cells were plated at a density of 5x104 cells/well on 6-well plates and grown overnight. The next day, the cells were treated with 5-Fu (100 μmol/L), and HT-29 cells without 5-Fu treatment were used as the control groups. Each treatment was performed in triplicate, and three different experiments were performed. After 96 h, the cells were harvested, counted, transferred to flow tubes, pelleted, and resuspended in 100 μl of fresh 1X Annexin binding buffer (0.01 M HEPES pH 7.4; 0.14 M NaCl; 2.5 mM CaCl_2_) plus Annexin V fluorescein isothiocyanate (FITC) and 7-AAD peridinin-chlorophyll protein (PerCP). After staining, the samples were analyzed by flow cytometry within 1 h using a FACSCanto system (BD). Annexin V^+^/7AAD^±^ cells were considered apoptotic cells. The proportion of apoptotic cells is expressed as a percentage of the total cell number acquired, excluding debris, and was analyzed using BD FACSDiva and FlowJo software (Tree Star, Inc., Ashland, OR, USA).

### Animal models

Four- to six-week-old Balb/c nude mice (body weight: 16-20 g) were purchased from the Experimental Animal Center, Institute of Laboratory Animal Sciences, China, and maintained in a specific pathogen-free (SPF) environment in accordance with the guidelines of the NIH (Guide for the Care and Use of Laboratory Animals, 1996) (23). The mice were subcutaneously injected with CRC cells (5×10^6^ cells in 100 uL of PBS/mouse, SEMA3F-overexpressing (SEMA3F OE) LoVo cells for the test groups, LoVo cells for the control groups, 10 nude mice in each group) in the left groin. Two weeks after tumor cell inoculation, animals with tumor sizes reaching approximately 50 mm^3^ received 5-Fu at 3 mg/kg by peritumoral injection every 48 h for 2 weeks. The mice were sacrificed, and the tumors were collected, half of which were fixed in 4% formalin and embedded in paraffin blocks. The remaining samples were snap-frozen in liquid nitrogen for histological studies in which five independent fields for each of the three tumors per group were evaluated. All of our animal studies were approved by the Institutional Animal Care and Use Committee of the Fuling Central Hospital of Chongqing City. Anesthetic procedures were used, and the animals did not suffer unduly during or after the experimental procedure.

### Statistical analyses

All the data were analyzed using GraphPad Prism software (version 8.0). The immunohistological staining scores were analyzed by two biostatisticians in the Department of Statistics at Army Medical University, China. The Kaplan-Meier method and log-rank test were performed for survival analysis. To analyze 5-year disease-free survival (DFS), events were defined as primary local-regional or distant tumor relapse. Descriptive and mean analyses were performed by one-way ANOVA (>3 groups) or Student’s t-test (between two groups). P values less than 0.05 were considered to indicate significance.

## Results

### MSC induced CRC cell chemotherapeutic resistance to 5-Fu *in vitro* by suppressing SEMA3F

**Figure 1:**
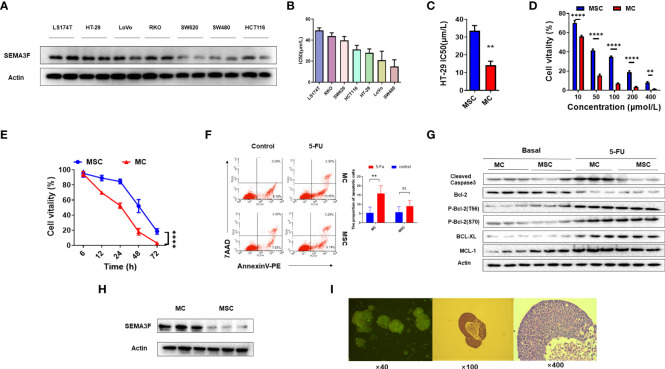
MSC exhibits resistance to 5-Fu. **(A)** Expression of SEMA3F in a series of human CRC cell lines (LS174T, HT-29, LoVo, RKO, SW620, SW480 and HCT-116 cells). **(B)** IC50 value of 5-Fu in a series of human CRC cell lines under MSC conditions. **(C)** IC50 value of 5-Fu in HT-29 cells under MSC and MC conditions. A significant difference in IC50 value was observed between the two groups (**P<0.01). **(D)** The viability of HT-29 cells under MSC and MC conditions treated with 5-Fu at various concentrations (10, 50, 100, 200 and 400 μmol/L). Statistical analysis was performed by two-way ANOVA, and a significant difference among the groups was observed (**P<0.01, ****P<0.0001). **(E)** The survival curve of HT-29 cells under MSC and MC conditions treated with 5-Fu (200 μmol/L) for 72 h (****P<0.0001). **(F)** Flow cytometry assay to detect the apoptosis rate of HT-29 cells under MSC and MC conditions. The apoptosis rates of HT-29 cells in MC group treated with 5-Fu (100 μmol/L) or not were 16.55% (LR 13.65%, UR 2.90%) and 9.27% (LR 9.18%, UR 0.09%) respectively, and in MSC group were 13.03% (LR 9.74%, UR 3.29%) and 11.06% (LR 7.63%, UR 3.43%) respectively. The experiment was repeated three times and the statistical histogram was shown. Statistical analysis was performed by two-way ANOVA, and a significant difference was observed under MC conditions (**P<0.01). **(G)** Western blot analysis of expression of the apoptosis-related molecules MCL-1, BCL-XL, BCL-2 and cleaved caspase3 in HT-29 cells before and after 5-Fu treatment. **(H)** Western blot analysis of the expression level of SEMA3F in HT-29 cells under MSC and MC conditions. **(I)** Tumor spheroids formed by three-dimensional culture.

Our previous study demonstrated that SEMA3F signaling may contribute to reversing chemotherapy resistance in CRC cells (21), so we first verified the expression of SEMA3F in a series of CRC cell lines: HCT-116, LS174T, HT-29, SW480, SW620, LoVo and RKO cells (**Figure 1A**). Several lines of evidence clearly indicate that MSC promotes chemotherapy resistance in tumor cells compared with monolayer cell culture (MC) (5, 10, 11, 26). To demonstrate the ability of MSC to enhance CRC cell resistance to 5-Fu, we investigated the IC50 values of 5-Fu in the above cell lines (**Figure 1B**). Because HT-29 cells expressed SEMA3F and had a medium IC50 value in MC, we compared the IC50 values of 5-Fu in HT-29 cells in MSC and MC groups; the IC50 values of 5-Fu in MSC group were substantially increased (P<0.01) (**Figure 1C**). Compared with MC group, the cell viability in MSC group increased significantly after treatment with 5-Fu at different concentrations for 24 h (P<0.01) (**Figure 1D**). A dose- and time-dependent reduction in cell viability was observed following treatment with increasing concentrations of 5-Fu in MSC and MC group. The cells were treated with 200 μmol/L 5-Fu, and cell viability was measured at different time points from 6 to 72 h. Similarly, the cell viability of MSC group was significantly increased at 12, 24 and 48 h compared to that of MC group (P<0.0001) (**Figure 1E**). These results indicated that MSC resulted in increased 5-Fu resistance in CRC cells.

We then evaluated the effects of MSC on HT-29 cell apoptosis. In MC group, the percentage of apoptotic cells increased after 5-Fu treatment (**Figure 1F**, P<0.01), but no similar phenomenon was observed in MSC group, suggesting that MSC resulted in 5-Fu resistance. Similarly, the expression of MCL-1, an anti-apoptotic gene, was significantly higher in MSC group than that in MC group (**Figure 1G**). In addition, the phosphorylation of BCL-2 at threonine 56 (phospho-Bcl-2-Thr56) in the two groups was no different, but the phosphorylation of BCL-2 at serine 70 (phospho-Bcl-2-Ser70) was decreased in MSC group. After 5-Fu chemotherapy, the phosphorylation of BCL-2 at both the Thr56 and Ser70 sites was increased, but there was no difference between the two groups. Furthermore, regardless of whether chemotherapy had been applied, the expression of cleaved caspase3 in MSC group was significantly lower than that in MC group (**Figure 1G**). These results showed that SEMA3F expression may restore the chemotherapeutic sensitivity of CRC cells to 5-Fu. Then, we compared the expression of SEMA3F in HT-29 cells between the MSC and MC groups and found that SEMA3F expression was significantly decreased in MSC group (**Figure 1H**). **Figure 1I** showed tumor spheroids formed by three-dimensional culture of cell line HT-29. These data suggested that MSC-induced chemotherapy resistance in CRC cells was probably due to the inhibition of SEMAF expression.

### SEMA3F improved the chemotherapeutic response of CRC cells to 5-Fu *in vitro*

**Figure 2:**
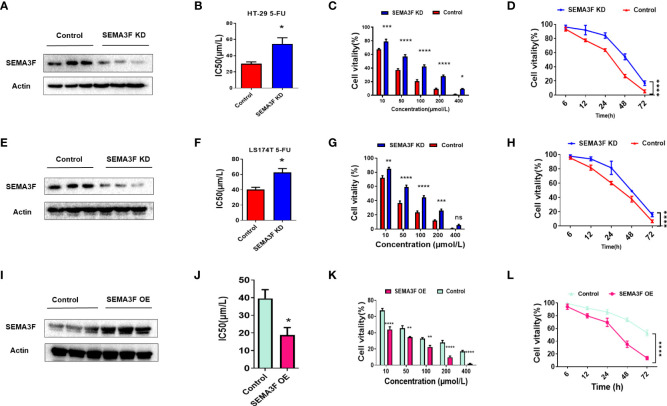
SEMA3F activity determines the chemotherapeutic response of CRC cells to 5-Fu *in vitro.* **(A)** SEMA3F was knocked down in HT-29 cells. **(B)** The IC50 value of 5-Fu in HT-29 cells under MSC conditions was higher in the SEMA3F KD group than in the control group (*P<0.05). **(C)** The viability rates of HT-29 cells with SEMA3F knockdown under MSC conditions were higher than that in the control groups upon treatment with 5-Fu at various concentrations (10, 50, 100, 200 and 400 μmol/L)(*P<0.05, ***P<0.001, ****P<0.0001). **(D)** The survival curve of HT-29 cells under MSC conditions treated with 5-Fu for 72 h (200 μmol/L) is shown, and HT-29 cell viability under MSC conditions was higher with SEMA3F knockdown than in the control groups (****P<0.0001). **(E)** SEMA3F was knocked down in LS174T cells. **(F)** The IC50 value of 5-Fu in LS174T cells under MSC conditions was higher in the SEMA3F KD groups than in the control groups (*P<0.05). **(G)** The viability of LS174T cells under MSC conditions was higher with SEMA3F knockdown than in the control groups upon treatment with 5-Fu at various concentrations (10, 50, 100, 200 and 400 μmol/L) (**P<0.01, ***P<0.001, ****P<0.0001). **(H)** The survival curve of LS174T cells under MSC conditions treated with 5-Fu (200 μmol/L) for 72 h is shown. LS174T cell viability under MSC conditions was higher with SEMA3F knockdown than in the control groups (****P<0.0001). **(I)** SEMA3F was overexpressed in LoVo cells. **(J)** The IC50 value of 5-Fu in LoVo cells under MSC conditions was lower in the SEMA3F OE group than in the control groups (*P<0.05). **(K)** The viability of LoVo cells with SEMA3F overexpression under MSC conditions was lower than that in the control groups upon treatment with 5-Fu at various concentrations (10, 50, 100, 200 and 400 μmol/L) (**P<0.01, ****P<0.0001). **(L)** The survival curve of LoVo cells under MSC conditions treated with 5-Fu (200 μmol/L) for 72 h is shown. The vitality of LoVo cells with SEMA3F OE under MSC conditions was lower than that in the control groups (****P<0.0001).

We further examined the effects of SEMA3F on the response of CRC cells to 5-Fu. Since the HT-29 and LS174T cell lines tend form multicellular spheroids in 3D culture, and the IC50 value of 5-Fu in HT-29 cells was lower while in LS174T cells was the highest, we chose these two cell lines for subsequent experiments. SEMA3F expression in the HT-29 and LS174T cell lines were knocked down, and the IC50 values of 5-Fu in the cells grown under MSC conditions and treated with 5-Fu were compared. We found that the IC50 values of SEMA3F KD group were higher than those of control group (P<0.05; **Figures 2A, B, E, F**). As the 5-Fu concentration increased and the treatment time was prolonged, the cell viability decreased gradually, but the viability of SEMA3F KD group was always higher than that of control group (P<0.05; **Figures 2C, D, G, H**). These results suggested that CRC cells were more resistant to 5-Fu when SEMA3F was knocked down. In contrast, when cells overexpressing SEMA3F were treated with 5-Fu (**Figure 2I**), the IC50 value was decreased (P<0.05, **Figure 2J**). In addition, the cell viability of SEMA3F OE group was always lower than that of control group, although a dose- and time-dependent reduction in cell viability was observed following treatment with 5-Fu (**Figures 2K, L**). These data demonstrated that SEMA3F activity enhanced the chemotherapeutic response of CRC cells to 5-Fu *in vitro*.

### SEMA3F overexpression enhanced the sensitivity of CRC cells to 5-Fu *in vivo*

**Figure 3:**
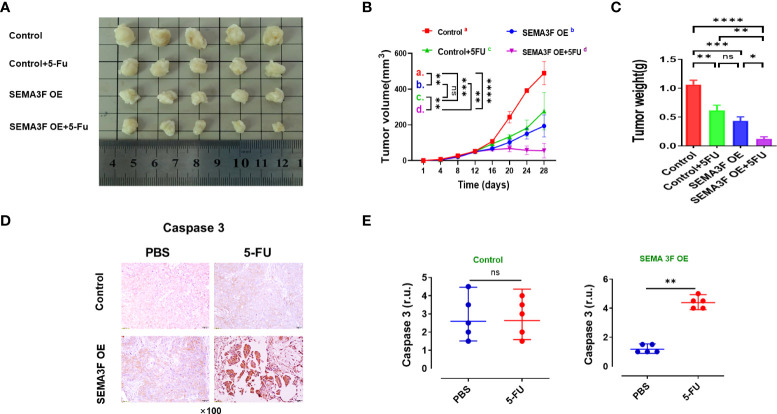
SEMA3F overexpression enhanced the sensitivity of CRC cells to 5-Fu *in vivo*. **(A)** Mice were subcutaneously injected with LoVo cells (SEMA3F OE LoVo cells for the test groups, LoVo cells for the control groups) in the left groin. Two weeks after tumor cell inoculation, animals were treated with 5-Fu or PBS by peritumoral injection every 48 h for 2 weeks.Then the mice were sacrificed, and the tumors were collected. **(B, C)** Statistical analysis of tumor volume and weight in the SEMA3F OE group and control group. (*P<0.05, **P<0.01, ***P<0.001, ****P<0.0001). **(D)** Immunohistochemical staining of caspase3 in CRC tissues from the SEMA3F OE groups and control groups treated with 5-Fu or PBS. **(E)** Statistical analysis of caspase3 in CRC tissues from the SEMA3F OE groups and control groups treated with 5-Fu or PBS (**P<0.01) ns, Not Statistically Significant.

To explore whether changes in SEMA3F expression could influence the sensitivity of CRC cells to 5-Fu *in vivo*, we injected SEMA3F OE LoVo cells into the left groin of nude mice and then treated them with 5-Fu or PBS. Mice treated with LoVo cells were used as the control groups. The volume and weight of the xenograft tumors in SEMA3F OE group were significantly lower than those in control group. After treatment with 5-Fu, the growth rate of xenograft tumors slowed down, especially in SEMA3F OE group (**Figures 3A−C**, P<0.05). Similarly, cleaved caspase3 tissue expression in SEMA3F OE group significantly increased with 5-Fu treatment. Regardless of whether 5-Fu treatment was applied or not, there was no difference in the expression of cleaved caspase3 in control group (**Figures 3D, E**). Consequently, these consistent data suggest that SEMA3F enhanced the sensitivity of CRC cells to 5-Fu and promoted apoptosis *in vivo*.

### SEMA3F enhances the sensitivity of CRC cells to 5-Fu by downregulating p27 activity

**Figure 4:**
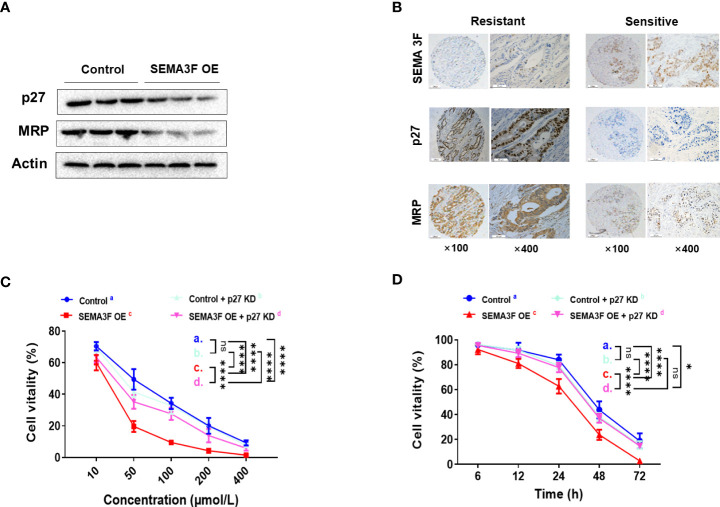
SEMA3F enhances the chemotherapeutic sensitivity of CRC cells to 5-Fu by downregulating p27. **(A)** Western blotting was used to assess the nuclear expression levels of p27 and MRP in the SEMA3F OE and control groups. **(B)** Immunohistochemical staining of SEMA3F, p27 and MRP in CRC patient tissues sensitive or resistant to chemotherapy. **(C)** The viability rates of LoVo cells with different expression levels of p27 and SEMA3F after treatment with 5-Fu at various concentrations (10, 50, 100, 200 and 400 μmol/L) are shown (****P<0.0001). **(D)** The survival curves of LoVo cells expressing different levels of p27 and SEMA3F after treatment with 5-Fu (200 μmol/L) for 72 h are shown (*P<0.05, ****P<0.0001). ns= Not Statistically Significant (P>0.05).

Our previous study demonstrated that SEMA3F signaling significantly reduced aVβ3 integrin expression to prevent chemotherapy resistance in CRC cells (21), and other studies have reported that p27 plays an important role in inducing drug resistance by increasing the expression of E-cadherin and integrin (8, 12). Thus, we investigated whether SEMA3F enhanced the sensitivity of CRC cells to 5-Fu by downregulating p27 expression. We compared the nuclear protein expression of p27 and multidrug resistance-associated protein (MRP) in SEMA3F OE and control LoVo cells under MSC. Significantly reduced nuclear expression of the p27 and MRP proteins was observed in the SEMA3F OE groups (**Figure 4A**). Immunohistochemical staining showed that in CRC patient samples, SEMA3F expression was positive(++) in chemotherapy-sensitive tissues while negative(-) in chemotherapy-resistant samples. On the contrary, the expression of p27 and MRP was weakly positive(+~++) in chemotherapy sensitive tissues, while strongly positive(+++) in chemotherapy resistant samples. (**Figure 4B**). We next asked whether we could enhance chemotherapy sensitivity by knocking down P27 expression in CRC cells. Thus, we employed p27-knockdown LoVo cells treated with 5-Fu and found that their cellular viability was not reduced upon 5-Fu treatment. Interestingly, P27 knockdown in LoVo cells did not decrease cellular viability with 5-Fu treatment; only in SEMA3F OE cells without P27 silencing was cellular viability significantly decreased (**Figures 4C, D**). Overall, these results demonstrated that SEMA3F did not enhance the sensitivity of CRC cells to 5-Fu when p27 expression was completely absent. It may be that the complete blockade of P27 expression caused the CRC cells to proliferate too fast. SEMA3F might enhance the chemotherapeutic sensitivity of CRC cells to 5-Fu by partially downregulating p27 activity.

### SEMA3F promotes p27 degradation by interacting with p27 and inducing p27 phosphorylation at Ser10

**Figure 5:**
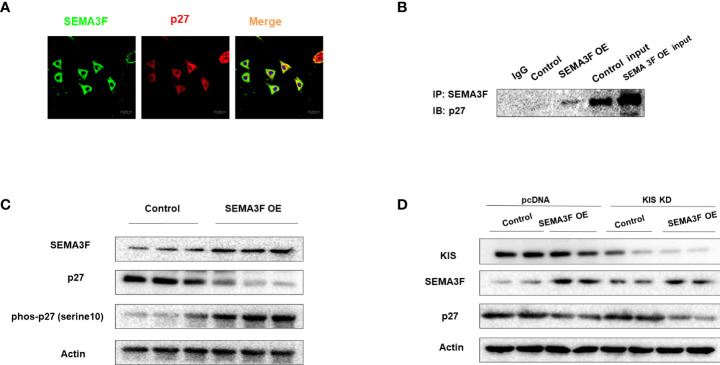
SEMA3F promotes p27 degradation by interacting with p27 and inducing its phosphorylation at Ser10. **(A)** Immunofluorescence analysis of SEMA3F (green) and p27 (red) in LoVo cells with SEMA3F OE. **(B)** The interaction between SEMA3F and p27 was assessed using an immunoprecipitation assay with LoVo cells with SEMA3F OE. **(C)** p27 and phosphorylated p27 expression levels in LoVo cells with SEMA3F OE and control cells were determined by Western blot analysis. **(D)** Western blot analysis was used to determine SEMA3F and p27 expression levels in the SEMA3F OE and control groups when the phosphorylation of p27 was or was not inhibited.

To explore the underlying interaction between SEMA3F and p27, we overexpressed SEMA3F in CRC cells under MSC conditions and employed these cells as a model to determine the interaction between SEMA3F and p27 using immunofluorescence analysis and immunoprecipitation. Immunofluorescence analysis showed that the expression of SEMA3F was negatively correlated with the nuclear expression of P27 (**Figure 5A**). Interestingly, p27 was primarily localized in the cytoplasm, not the nucleus. This finding may explain the ability of SEMA3F to downregulate P27 nuclear expression. Furthermore, the coimmunoprecipitation results suggested an interaction between SEMA3F and p27 (**Figure 5B**). Additionally, it was previously confirmed that p27 is phosphorylated much earlier at Ser10 than at other phosphorylation sites; this process is required for p27 nuclear export and accounts for approximately 70% of the total phosphorylation of this protein (27–29). Therefore, we measured the phosphorylation of p27 at Ser10 when SEMA3F was overexpressed. We found that along with SEMA3F expression, the phosphorylation of p27 at Ser10 was increased (**Figure 5C**). Human KIS (hKIS) has been shown to mediate the phosphorylation of p27 at Ser10 (30). Thus, we measured the change in p27 at different SEMA3F expression levels when hKIS was knocked down in CRC cells under MSC conditions. The p27 expression level in the nucleus was no different in the control and SEMA3F OE groups. However, when hKIS expression was normal, p27 nuclear expression was decreased in the SEMA3F OE groups compared with the control groups (**Figure 5D**). These results indicate that SEMA3F promotes p27 phosphorylation at Ser10 to degrade p27 and decrease its nuclear export.

### CRC patients with high SEMA3F expression are more sensitive to 5-Fu chemotherapy than those with low SEMA3F expression

**Figure 6:**
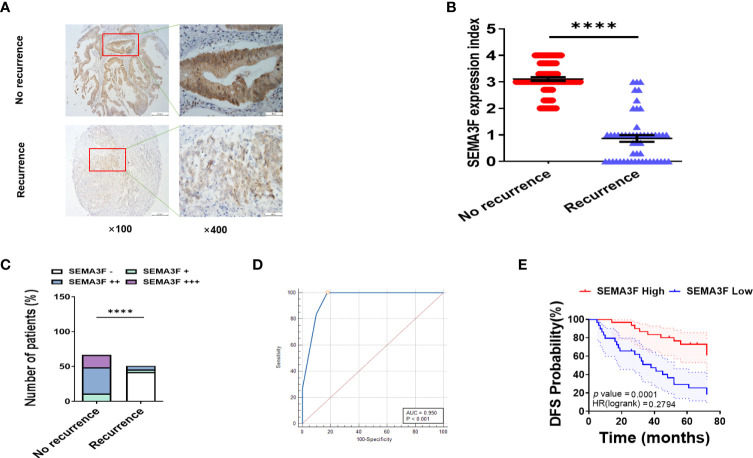
CRC patients with high SEMA3F expression are more sensitive to 5-Fu chemotherapy than patients with low expression. **(A)** Immunohistochemical staining of SEMA3F was carried out in tissues from patients with recurrent CRC and patients with no recurrence. **(B)** The SEMA3F expression indexes in the recurrence and no recurrence groups are shown (****P<0.0001). **(C)** Statistical analysis of immunohistochemical staining data for SEMA3F in patients with recurrent and nonrecurrent CRC (****P<0.0001). **(D)** ROC curve reveals that SEMA3F expression is a predictor for 5-Fu sensitivity. **(E)** Statistical analysis of 6-year disease-free survival in patients with high SEMA3F expression and low SEMA3F expression (HR=0.28, 95% CI: 0.14-0.56, P=0.0001).

We further confirmed the above findings in CRC patients. The expression of SEMA3F was detected by immunohistochemistry in postoperative tissue samples from patients with recurrent and nonrecurrent CRC who received adjuvant chemotherapy and were followed up for six years. All patients had received 5-Fu-based adjuvant chemotherapy for 3 to 6 months, with an average treatment time of 4.5 ± 1.5 months. Because adjuvant chemotherapy for colon cancer is 5-Fu monotherapy or 5-Fu plus oxaliplatin (based on the patient’s postoperative staging status), the chemotherapy regimen was consistent and not subject to interference by irinotecan, antiangiogenic therapy, or molecular targeted therapy. The results showed that SEMA3F staining was significantly stronger in the tissue samples from patients with nonrecurrent CRC than in those from patients with recurrent CRC (**Figure 6A**). The SEMA3F expression index was markedly higher in the group without recurrence than in the recurrence group (**Figure 6B**). Furthermore, SEMA3F expression of majority of no recurrence tissue samples (56/67) were strongly positive (++~+++), but most of the recurrent tissue samples (46/51) were negative(-) or weakly positive(+) (**Figure 6C**). ROC curve further revealed that SEMA3F expression is a predictor for 5-Fu sensitivity(**Figure 6D**). According to the ROC curve, we chose “++” as the cut off value, defined “- ~ +” as “low expression” and “++~+++” as “high expression”. These patients were grouped based on this standard and followed up for 6 years to explored the prognostic value of SEMA3F in CRC patients. The median DFS of the low SEMA3F expression group was only 38 months, but the median DFS of the high SEMA3F expression group was still undefined (HR=0.28, 95% CI: 0.14-0.56, P=0.0001, **Figure 6E**). The above results suggested that CRC patients with high SEMA3F expression are more sensitive to 5-Fu chemotherapy than those with low SEMA3F expression.

## Discussion

In this study, we found that SEMA3F expression was downregulated and that the IC50 value of 5-Fu in CRC cells was substantially increased in the MSC groups. Our previous study demonstrated that SEMA3F expression significantly reduced αVβ3 integrin expression levels in CRC cells to block chemotherapy resistance (21). Hence, we believe that SEMA3F may prevent tumor microenvironment-mediated MCR.

p27 plays an important role in inducing chemotherapy resistance in multicellular spheroids by increasing E-cadherin and integrin expression levels. Previous studies on tumor multicellular drug resistance have mainly focused on enhanced cell adhesion, resulting in decreased penetration of chemotherapeutic drugs and the entrance of tumor cells into a quiescent state. When HT29 human colorectal cancer cells converged, the expression of p27 increased, and the cells accumulated in the G0/G1 phase of the cell cycle. Nonfused HT29 cells overexpressing p27 were also more resistant to anticancer drugs (31). However, this study did not use a multicellular spheroid model. The core area of HCT116 multicellular spheroids was negative for the proliferation marker Ki67 but positive for p27. One possible mechanism for the upregulation of p27 is downregulation of the ERK signal, which is observed in multicellular spheroids (32). Our research focuses on how the SEMA3F signal inhibits P27 function to reverse tumor multicellular drug resistance and its mechanism. In this study, we upregulated SEMA3F expression *in vitro* and observed increased sensitivity to 5-Fu in CRC cells in the MSC groups; the same result was found *in vivo*, and CRC cell apoptosis was induced. When we overexpressed SEMA3F in CRC cells, CRC multicellular spheroids showed significantly decreased p27 nucleoprotein expression and increased chemosensitivity. Interestingly, we found that P27 knockdown did not affect the response of the CRC cells under MSC to 5-Fu, and only SEMA3F OE cells without P27 silencing exhibited increased chemosensitivity. It may be that the complete blockade of P27 expression caused the CRC cells to proliferate too fast. This phenomenon is consistent with the clinical treatment of refractory lymphoma with high proliferative activity (33). This indicates that SEMA3F might enhance the chemotherapeutic sensitivity of CRC cells to 5-Fu by partially downregulating p27 activity. In 5-Fu-sensitive CRC patient samples, we found that p27 and MRP were barely detected, whereas SEMA3F was highly expressed. The molecular mechanism by which SEMA3F signals reverse MCR in CRC cells remains unknown.

Moreover, when we upregulated SEMA3F expression in CRC cells, p27 was primarily localized in the cytoplasm but not in the nucleus. These data implied that SEMA3F prevents the nuclear localization of p27 to promote cell cycle progression. How does SEMA3F induce p27 cytoplasmic translocation? We found that SEMA3F stimulates the phosphorylation of p27 at Ser10. Several previous studies have shown that the phosphorylation of p27 at Ser10 induces its export to the cytoplasm and promotes its degradation at G1 phase (34, 35). These results indicated that SEMA3F promoted p27 phosphorylation at Ser10, leading to its export from the nucleus to the cytoplasm. They also suggested that SEMA3F increased p27 degradation, rather than its proteolysis, to induce tumor cells to enter the cell cycle. These results reveal for the first time that SEMA3F mediates p27 degradation by inducing p27 phosphorylation at Ser10.

How does SEMA3F, as a secretory protein, affect signals in the nucleus? In a study of neural development, SEMA3A was found to inhibit the growth of axonal growth cones through the phosphorylation of CDK-5 and glycogen synthase kinase-3 (GSK3) (36). SEMA3A and SEMA3F can also suppress rapamycin complex (mTORC) signaling (37, 38). SEMA6A bound to Fas-associated protein with death domain (FADD) to induce cytosol-induced apoptosis (39). Recently, it has been reported that retinoic acid receptor-related orphan receptor α (ROR α) (40), Achaete scute-like 2 (ASCL2) (41) and DNA inhibitor binding/differentiation 2 (Id2) (42) bind the promoter region of SEMA3F in colon cancer. These three transcription factors promote or inhibit SEMA3F transcription to affect its expression. In this study, SEMA3F was found to cause the cytoplasmic translocation of P27. However, whether cytoplasmic P27 translocation is caused by unsecreted SEMA3F in the cytoplasm and how secreted proteins affect intracellular signals needs to be further studied.

## Conclusions

In conclusion, our data show that SEMA3F reverses CRC cell and patient chemotherapy resistance by inducing the phosphorylation of p27 at Ser10 to modulate the subcellular localization of p27. Our findings present a novel mechanism underlying the role of SEMA3F in the chemosensitivity of CRC cells and suggest a potential therapeutic strategy for CRC.

## Data availability statement

The original contributions presented in the study are included in the article/supplementary material. Further inquiries can be directed to the corresponding author.

## Ethics statement

The animal study was reviewed and approved by the Animal Care and Use Committee of Fuling Central Hospital of Chongqing City, China.

## Author contributions

MC and QZ designed the study. QZ, MT and HM prepared the figures and wrote the article. MT and HM performed all of the gene transfection experiments, Western blotting, and data analysis. XF and CW generated the CRC cell multicellular spheroid cultures and monolayer cell clusters and carried out gene knockout. YLi, XH and XQ performed the immunohistochemical and immunofluorescence assays. RLv and GZ performed the flow cytometry and apoptosis assays. JW and MZ performed the chemotherapeutic and chemosensitivity assays. GX performed the coimmunoprecipitation assays. QH and YLo performed the animal experiments. ZW provided valuable recommendations, contributing to the study design and data analysis. RLi performed the follow-up. All authors contributed to the article and approved the submitted version.

## Funding

This work was supported by grant number zdxk201920 from Chongqing Municipal Health Commission, grant number cstc2019jcyj-msxmX0711 from the Natural Science Foundation of Chongqing Municipal Science and Technology Commission (to QZ) and grant number ZY201802003 from the Chongqing medical scientific research project (Joint project of Chongqing Health Commission and Science and Technology Bureau)(to MT).

## Acknowledgments

We would like to thank the referees and editor, who provided outstanding advice and helped us build the concepts presented in this work. We thank Dr. Juanjuan Ou, Department of Oncology, Southwest Hospital, Army Medical University, Chongqing, China, for her excellent technical assistance and help in editing this manuscript. The authors thank Dr. Feng Wu, Institute of Pathology of Army Medical University, for help in editing this manuscript. We thank Wei Sun of the Central Laboratory of Army Medical University for her excellent technical assistance in confocal laser scanning microscopy.

## Conflict of interest

The authors declare that the research was conducted in the absence of any commercial or financial relationships that could be construed as a potential conflict of interest.

## Publisher’s note

All claims expressed in this article are solely those of the authors and do not necessarily represent those of their affiliated organizations, or those of the publisher, the editors and the reviewers. Any product that may be evaluated in this article, or claim that may be made by its manufacturer, is not guaranteed or endorsed by the publisher.

## References

[1] Siegel RL Miller KD Fuchs HE Jemal A. Cancer statistics, 2022. Ca-Cancer J Clin (2022) 72(1):7–33. doi: 10.3322/caac.21708 35020204

[2] Rebecca LS Kimberly DM Ann GS Stacey AF Lynn FB Joseph CA. Colorectal cancer statistics, 2020. Ca-Cancer J Clin (2020) 70(3):145–64. doi: 10.3322/caac.21601

[3] L. A K. T B. A S-P. X D. S A. E. A mechanically active heterotypic e-cadherin/N-cadherin adhesion enables fibroblasts to drive cancer cell invasion. Nat Cell Biol (2017) 19(3):224–37. doi: 10.1038/ncb3478 PMC5831988 28218910

[4] Dong Y Tan OL Loessner D Stephens C Walpole C Boyle GM. Kallikrein-related peptidase 7 promotes multicellular aggregation *via* the α5β1 integrin pathway and paclitaxel chemoresistance in serous epithelial ovarian carcinoma. Cancer Res (2010) 70(7):2624–33. doi: 10.1158/0008-5472.CAN-09-3415 20332224

[5] Kang H-G Jenabi JM Zhang J Keshelava N Shimada H May WA. E-cadherin cell-cell adhesion in Ewing tumor cells mediates suppression of anoikis through activation of the ErbB4 tyrosine kinase. Cancer Res (2007) 67(7):3094–105. doi: 10.1158/0008-5472.CAN-06-3259 PMC3906735 17409416

[6] D PWB L X S F vdG H Z J E B. Somatic inactivation of e-cadherin and p53 in mice leads to metastatic lobular mammary carcinoma through induction of anoikis resistance and angiogenesis. Cancer Cell (2006) 10(5):437–49. doi: 10.1016/j.ccr.2006.09.013 17097565

[7] Seifert A Posern G. Tightly controlled MRTF-a activity regulates epithelial differentiation during formation of mammary acini. Breast Cancer Res (2017) 19(1):68. doi: 10.1186/s13058-017-0860-3 28592291 PMC5463372

[8] Tsytlonok M Sanabria H Wang Y Felekyan S Hemmen K. Phillips AH et al: Dynamic anticipation by Cdk2/Cyclin a-bound p27 mediates signal integration in cell cycle regulation. Nat Commun (2019) 10(1):1676. doi: 10.1038/s41467-019-09446-w 30976006 PMC6459857

[9] Bi E Li R Bover L Li H Su P Ma X. E-cadherin expression on multiple myeloma cells activates tumor-promoting properties in plasmacytoid DCs. J Clin Invest (2018) 128(11):4821–31. doi: 10.1172/JCI121421 PMC6205396 30277474

[10] LaRue KEA Khalil M Freyer JP. Microenvironmental regulation of proliferation in multicellular spheroids is mediated through differential expression of cyclin-dependent kinase inhibitors. Cancer Res (2004) 64(5):1621–31. doi: 10.1158/0008-5472.CAN-2902-2 14996720

[11] Galateanu B. Hudita A. Negrei C. Ion R.M. Costache M. Stan M.. Impact of multicellular tumor spheroids as an *in vivo*−like tumor model on anticancer drug response. Int J Oncol (2016) 48(6):2295–302. doi: 10.3892/ijo.2016.3467 PMC4867843 27035518

[12] Das RK Huang Y Phillips AH Kriwacki RW Pappu RV. Cryptic sequence features within the disordered protein p27Kip1 regulate cell cycle signaling. Proc Natl Acad Sci U S A (2016) 113(20):5616–21. doi: 10.1073/pnas.1516277113 PMC4878473 27140628

[13] Carduner L Picot CR Leroy-Dudal J Blay L Kellouche S Carreiras F. Cell cycle arrest or survival signaling through alpha v integrins, activation of PKC and ERK1/2 lead to anoikis resistance of ovarian cancer spheroids. Exp Cell Res (2014) 320(2):329–42. doi: 10.1016/j.yexcr.2013.11.011 24291221

[14] Liu Y Lv J Liang X Yin X Zhang L Chen D. Fibrin stiffness mediates dormancy of tumor-repopulating cells *via* a Cdc42-driven Tet2 epigenetic program. Cancer Res (2018) 78(14):3926–37. doi: 10.1158/0008-5472.CAN-17-3719 29764867

[15] Doci C Mikelis C Lionakis M Molinolo A Gutkind J. Genetic identification of SEMA3F as an antilymphangiogenic metastasis suppressor gene in head and neck squamous carcinoma. Cancer Res (2015) 75(14):2937–48. doi: 10.1158/0008-5472.CAN-14-3121 PMC4538958 25952650

[16] Bielenberg DR Hida Y Shimizu A Kaipainen A Kreuter M Kim CC. Semaphorin 3F, a chemorepulsant for endothelial cells, induces a poorly vascularized, encapsulated, nonmetastatic tumor phenotype. J Clin Invest (2004) 114(9):1260–71. doi: 10.1172/JCI200421378 PMC524226 15520858

[17] Futamura M Kamino H Miyamoto Y Kitamura N Nakamura Y Ohnishi S. : Possible role of semaphorin 3F, a candidate tumor suppressor gene at 3p21.3, in p53-regulated tumor angiogenesis suppression. Cancer Res (2007) 67(4):1451–60. doi: 10.1158/0008-5472.CAN-06-2485 17308083

[18] Bollard J Massoma P Vercherat C Blanc M Lepinasse F Gadot N. The axon guidance molecule semaphorin 3F is a negative regulator of tumor progression and proliferation in ileal neuroendocrine tumors. Oncotarget (2015) 6(34):36731–45. doi: 10.18632/oncotarget.5481 PMC4742207 26447612

[19] Ou J Wei X Peng Y Zha L Zhou R Shi H. Neuropilin-2 mediates lymphangiogenesis of colorectal carcinoma *via* a VEGFC/VEGFR3 independent signaling. Cancer Lett (2015) 358(2):200–9. doi: 10.1016/j.canlet.2014.12.046 25543087

[20] Wu F Zhou Q Yang J Duan G Ou J Zhang R. Endogenous axon guiding chemorepulsant semaphorin-3F inhibits the growth and metastasis of colorectal carcinoma. Clin Cancer Res (2011) 17(9):2702–11. doi: 10.1158/1078-0432.CCR-10-0839 21349996

[21] Zheng C Zhou Q Wu F Peng Q Tang A Liang H. Semaphorin3F down-regulates the expression of integrin alpha(v)beta3 and sensitizes multicellular tumor spheroids to chemotherapy via the neuropilin-2 receptor *in vitro* . Chemotherapy (2009) 55(5):344–52. doi: 10.1159/000232449 19657188

[22] Patrick N Sophie K Bruno C Valérie C Harry AD Dominique B. Semaphorin SEMA3F has a repulsing activity on breast cancer cells and inhibits e-cadherin-mediated cell adhesion. Neoplasia (2005) 7(2):180–9. doi: 10.1593/neo.04478 PMC1501131 15802023

[23] Bahnassy AA Helal TE-A El-Ghazawy IMH Samaan GF Galal el-Din MM Abdellateif MS. The role of e-cadherin and Runx3 in helicobacter pylori-associated gastric carcinoma is achieved through regulating P21waf and P27 expression. Cancer Genet (2018) 228(1):64–72. doi: 10.1016/j.cancergen.2018.08.006 30553475

[24] Jolly MK Ware KE Xu S Gilja S Shetler S Yac Y. E-cadherin represses anchorage-independent growth in sarcomas through both signaling and mechanical mechanisms. Mol Cancer Res (2019) 17(6):1391–402. doi: 10.1158/1541-7786.MCR-18-0763 PMC6548594 30862685

[25] Catalano A Caprari P Rodilossi S Betta P Castellucci M Casazza A. Cross-talk between vascular endothelial growth factor and semaphorin-3A pathway in the regulation of normal and malignant mesothelial cell proliferation. FASEB J (2004) 18(2):358–60. doi: 10.1096/fj.03-0513fje 14656993

[26] Powan P Luanpitpong S He X Rojanasakul Y Chanvorachote P. Detachment-induced e-cadherin expression promotes 3D tumor spheroid formation but inhibits tumor formation and metastasis of lung cancer cells. Am J Physiol (2017) 313(5):C556–66. doi: 10.1152/ajpcell.00096.2017. Physiology FoPSCUBT. PMC5792170 28931539

[27] Hnit SST Xie CL Yao M Holst J Bensoussan A De Souza P. p27(Kip1) signaling: Transcriptional and post-translational regulation. Int J Biochem Cell Biol (2015) 68:9–14. doi: 10.1016/j.biocel.2015.08.005 26279144

[28] Li Y Nakka M Kelly AJ Lau CC Krailo M Barkauskas DA. p27 is a candidate prognostic biomarker and metastatic promoter in osteosarcoma. Cancer Res (2016) 76(13):4002–11. doi: 10.1158/0008-5472.CAN-15-3189 PMC4930684 27197201

[29] Hyunho Y Minsoon K Kibeom J Miyoung S Besser A Xue X. p27 transcriptionally coregulates cJun to drive programs of tumor progression. Proc Natl Acad Sci U S A (2019) 116(14):7005–14. doi: 10.1073/pnas.1817415116 PMC6452716 30877256

[30] Wang H Lee W. Molecular mechanisms underlying progesterone-induced cytoplasmic retention of p27 in breast cancer cells. J Steroid Biochem Mol Biol (2018) 183:202–9. doi: 10.1016/j.jsbmb.2018.06.015 29959971

[31] Dimanche-Boitrel M-T Micheau O Hammann A Haugg M Eymin B Chauffert B. Contribution of the cyclin-dependent kinase inhibitor p27KIP1 to the confluence-dependent resistance of HT29 human colon carcinoma cells. Int J Cancer (1998) 77(5):796–802. doi: 10.1002/(SICI)1097-0215(19980831)77:5<796:AID-IJC20>3.0.CO;2-Z 9688315

[32] Zhang X de Milito A Olofsson MH Gullbo J D'Arcy P Linder S. Targeting mitochondrial function to treat quiescent tumor cells in solid tumors. Int J Mol Sci (2015) 16(11):27313–26. doi: 10.3390/ijms161126020 PMC4661878 26580606

[33] Sehn LH Herrera AF Flowers CR Kamdar MK McMillan A Hertzberg M. Polatuzumab vedotin in relapsed or refractory diffuse Large b-cell lymphoma. J Clin Oncol (2020) No.2):155–65. doi: 10.1200/JCO.19.00172 PMC7032881 31693429

[34] Théard D Raspe MA Kalicharan D Hoekstra D Van IJzendoorn SCD. Formation of e-cadherin/β-catenin-based adherens junctions in hepatocytes requires serine-10 in p27(Kip1). Mol Biol Cell (2008) 19(4):1605–13. doi: 10.1091/mbc.E07-07-0661 PMC2291403 18272788

[35] Li Z Tao Y Wang X Jiang P Li J Peng M. Tumor-secreted exosomal miR-222 promotes tumor progression *via* regulating P27 expression and re-localization in pancreatic cancer. Cell Physiol Biochem (2018) 51(2):610–29. doi: 10.1159/000495281 30458449

[36] Perlini L Szczurkowska J Ballif B Piccini A Sacchetti S Giovedi S. Synapsin III acts downstream of semaphorin 3A/CDK5 signaling to regulate radial migration and orientation of pyramidal neurons *In vivo* . Cell Rep (2015) 11(2):234–48. doi: 10.1016/j.celrep.2015.03.022 PMC4405008 25843720

[37] Nakayama H Bruneau S Kochupurakkal N Coma S Briscoe DM Klagsbrun M. Regulation of mTOR signaling by semaphorin 3F-neuropilin 2 interactions *in vitro* and *In vivo* . Sci Rep (2015) 15:11789. doi: 10.1038/srep11789 PMC4496725 26156437

[38] Yamada D Kawahara K Maeda T. mTORC1 is a critical mediator of oncogenic Semaphorin3A signaling. Biochem Biophys Res Commun (2016) 476(4):475–80. doi: 10.1016/J.BBRC.2016.05.147 27246732

[39] Shen C-Y Chang Y-C Chen L-H Lin W-C Lee Y-H Yeh S-T. The extracellular SEMA domain attenuates intracellular apoptotic signaling of semaphorin 6A in lung cancer cells. Oncogenesis (2018) 7. doi: 10.1038/s41389-018-0105-z PMC6281666 30518871

[40] Xiong G Wang C Evers BM Zhou BP Xu R. RORα suppresses breast tumor invasion by inducing SEMA3F expression. Cancer Res (2012) 72(7):1728–39. doi: 10.1158/0008-5472.CAN-11-2762 PMC3319846 22350413

[41] Zhou Z Rao J Yang J Wu F Tan J Xu S. SEMA3F prevents metastasis of colorectal cancer by PI3K-AKT-dependent down-regulation of the ASCL2-CXCR4 axis. J Pathol (2015) 236(4):467–78. doi: 10.1002/path.4541 25866254

[42] Coma S Amin DN Shimizu A Lasorella A Iavarone A Klagsbrun M. Id2 promotes tumor cell migration and invasion through transcriptional repression of semaphorin 3F. Cancer Res (2010) 70(9):3823–32. doi: 10.1158/0008-5472.CAN-09-3048 PMC2862101 20388805

